# Understanding the effect of component proportions on disease control in two-component cultivar cereal mixtures using a pathogen dispersal scaling hypothesis

**DOI:** 10.1038/s41598-023-31032-w

**Published:** 2023-03-11

**Authors:** Adrian C. Newton, Peter Skelsey

**Affiliations:** 1 Ecological Sciences, The James Hutton Institute, Invergowrie, Dundee, DD2 5DA Scotland, UK; 2 Information and Computational Sciences, The James Hutton Institute, Invergowrie, Dundee, DD2 5DA Scotland, UK

**Keywords:** Computational biology and bioinformatics, Plant sciences

## Abstract

A field experiment was carried out to determine the importance of component cultivar proportions to spring barley mixture efficacy against rhynchosporium or scald symptoms caused by the splash-dispersed pathogen *Rhynchosporium commune*. A larger effect than expected was observed of small amounts of one component on another for reducing disease overall, but relative insensitivity to proportion as amounts of each component become more similar. An established theoretical framework, the ‘Dispersal scaling hypothesis’, was used to model the expected effect of mixing proportions on the spatiotemporal spread of disease. The model captured the unequal effect of mixing different proportions on disease spread and there was good agreement between predictions and observations. The dispersal scaling hypothesis therefore provides a conceptual framework to explain the observed phenomenon, and a tool to predict the proportion of mixing at which mixture performance is maximized.

## Introduction

Cultivar mixtures are a means to improve yield above the mean of their component cultivars, and to enhance yield stability^1^. This is achieved through processes of compensation, complementation and facilitation^2^ resulting in improved resource use efficiency. Epidemic control may be further enhanced by spatial exploitation of specific resistance genes in different mixture components in relation to analysis of pathogen populations as well as more direct means of slowing the epidemic with some pathogens^3^. This is achieved through spatial components: the barrier effect of resistant cultivars and dilution of susceptible cultivars, and the induced resistant effect from the avirulent interactions preventing normally susceptible pathogen genotypes infecting^4^. The advantage of a mixture increases with the mean level of specific resistance of the component cultivars in barley against powdery mildew^5^. Partial resistance, if race non-specific, should not result in epidemic reduction if the host–pathogen interactions were purely due to specificity. However, even with highly specific obligate biotroph pathogens such as the powdery mildews on cereals, there are clearly other interactions taking place too^6,7^. Furthermore, mixtures can be highly effective for reducing epidemics in pathogens that are generally race non-specific such as several splash-dispersed pathogens^8–11^. These may be traits that affect canopy microclimate or the distance or tortuocity of splash dispersal pathways. Specific traits such as plant height, leaf habit and other aspects of canopy architecture have been studied to understand and model epidemic progress including the effect on pathogen dispersal by rain^12–16^. In practice we find that spatial connectivity of components in the mixture may be important also as coarse granularity has been shown to increase yield and reduce disease more than homogeneous mixing^17^.

With several traits that can contribute to mixture efficacy for both yield and epidemic control, it may require many different cultivars to bring them all together. These cultivars may contribute also non-beneficial or deleterious traits for pathogen control, yield or quality. Furthermore, we know little about the magnitude of effect of each trait and whether they contribute proportionately to overall efficacy for any given outcome, although some of this can be calculated in extensive full factorial trialling of monocultures and all their component combinations^11^. If trait contributions are disproportionate where more effect is observed than would be expected from the proportion in the mixture, particularly if threshold values can achieve much of the effect, then each component of a mixture could be included in different proportions depending on their response. This might give the option of including cultivars in small proportions that are not desirable as the main yield or quality component but that have some other highly expressed trait that is very beneficial in its expression in the mixture.

**Figure 1:**
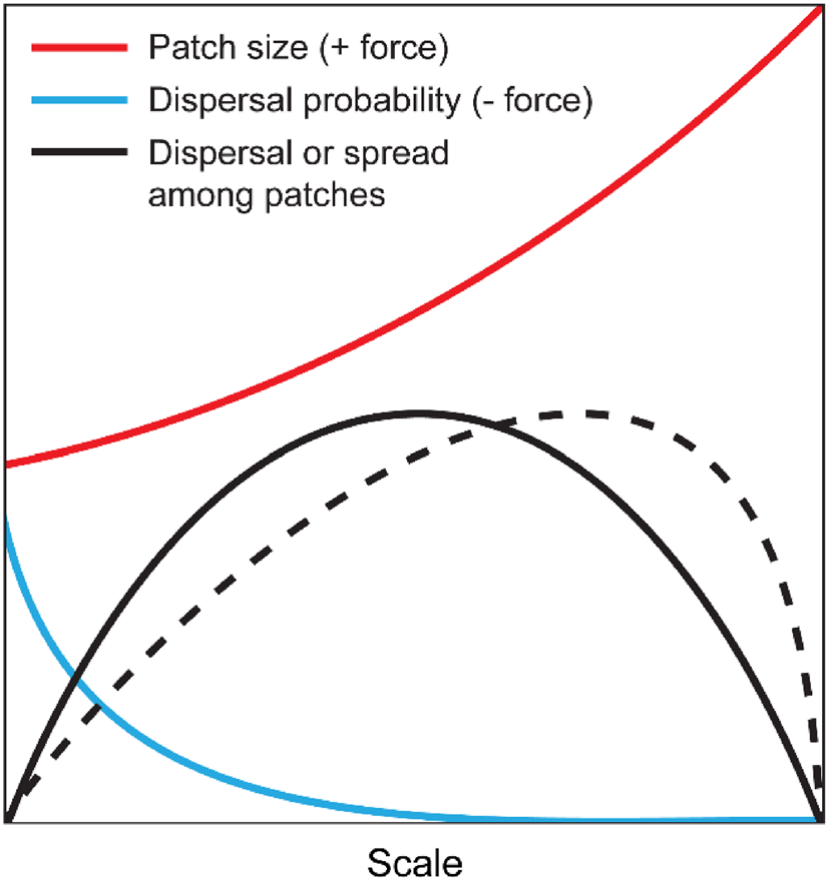
Conceptual representation of the dispersal scaling hypothesis (DSH). Scaling a heterogeneous or patchy environment relative to the gap-crossing abilities of a species produces antagonistic ‘dispersal forces’ that result in a unimodal scaling relationship for dispersal and spatiotemporal spread. The amount, quality, and distribution of habitat patches in the landscape can introduce asymmetry in the scaling relationship (dashed line).

Models to explore resistance deployment strategies can be spatially implicit^18–25^ or spatially explicit^26–34^; the former includes assumptions on how spatial structures affect dynamics, whereas the latter utilizes explicit representations of geographic space and spatial entities (see Rimbaud et al.^35^ for a recent, comprehensive review of relevant modelling studies). In this study we use a spatially implicit approach to negate the computer expense of representing individual plants explicitly, and to produce a more tractable and interpretable model. Specifically, we utilise an ecological theory—the dispersal scaling hypothesis (DSH)—to model the expected effects of spatial heterogeneity within crops on spatiotemporal spread of disease, i.e., the aforementioned barrier, dilution, and connectivity effects of mixtures on reduction of disease. The DSH posits a unimodal (‘humpbacked’) scaling relationship between the magnitude of dispersal (the number of dispersing agents moving between patches) and the grain size (scale of patchiness) of a heterogeneous habitat distribution^36–38^. Conceptually, we can envision this relationship emerging as a consequence of antagonistic ‘dispersal forces’ operating at different scales (Fig. 1). As grain size increases, the number of dispersal agents within each (larger) patch increases, and the larger patches also act as bigger ‘targets’ or are perceived to be more attractive to dispersers. This produces a ‘positive dispersal force’ that results in increased dispersal (Fig. 1; red line). Concomitantly, the distances between patches increase, which decreases the probability of movement among patches, resulting in an accompanying ‘negative dispersal force’ (Fig. 1; blue line). Ultimately, this produces a unimodal relationship between dispersal and spatial grain size, where dispersal is maximised when the trade-off between these competing forces is optimized (Fig. 1; black line). This maximum will occur when the scale of spatial heterogeneity is neither too fine nor too coarse relative to the gap-crossing abilities of the organism; that is, at an intermediate range of grain sizes. The exact grain size will depend on other aspects of spatial heterogeneity, such as the amount, quality, and distribution of habitat patches in the landscape. These other aspects of heterogeneity can skew the unimodal distribution due to scale-dependent effects on the trade-off between antagonistic dispersal forces (Fig. 1; dashed line). This scaling relationship was also demonstrated for spatiotemporal spread of numerous pests and diseases in a simulation study, i.e., for multiple generations of population growth and dispersal^37^.

Previous mixtures studies have generally used equal proportion of the components but a few have looked at the effects of different proportions but generally no less than 25% of any one^39–41^. In the context of the current study, we investigate the full range of proportions and hypothesise that mixture performance will be maximised at an intermediate proportion (grain) of mixing, resulting in a ‘u-shaped’ unimodal scaling distribution of mixture performance versus mixing proportion. The expected shape of the scaling relationship is inverted compared to Fig. 1 as the response variable, mixture performance, is not an absolute amount of disease but a decrease in disease relative to the mean of the pure stands. It is further expected that differential trait effects (i.e., patch quality) will introduce asymmetry in the scaling distribution (Fig. 1; dashed line). As an experimental system we use spring barley cultivars of commercial relevance and focus on the main disease that can occur naturally in our research farm environment, ‘rhynchosporium’, ‘scald’ or barley leaf blotch, caused by the hemi-biotrophic splash-dispersed pathogen *Rhynchosporium commune* and previously reported to show disease reduction and yield gain in mixtures^11^.

## Materials and methods

### Field trials

The field trial was conducted at Mylnefield (56° 27′ 19.3″ N 3° 04′ 08.3″ W), near Dundee, Scotland in sandy loam soil with a mean June temperature is 13 °C and the annual rainfall is 722 mm and 1426 h of sunshine. Commercial seed treated with a standard seed dressing were weighed according to proportion adjusted by thousand grain weight and thoroughly mixed. Seed was sown in 8-row plots at a sowing density of 360 seed/m^2^ in April using a Hege seed plot drill. Plots were 1.55 m wide by 6.0 m long, reduced to 4.8 m with plot definitions and three replicates were sown in a split-plot design. Phosphate (P_2_O_5_) and potassium (K_2_O) (0:30:20) were applied at sowing at a rate of 70 and 105 kg/ha respectively at sowing followed by two top-dressings of nitrogen (30:0:0) totalling 250 kg/ha and standard pre- and post-emergence herbicides were applied. All methods were performed in accordance with the relevant guidelines and regulations.

### Disease assessments

All plots were assessed for disease regularly and scored approximately every 1–2 weeks from first occurrence above trace levels on a 1–9 whole plot scale where 1 is no disease and 9 is 100% symptoms^42,43^. Scores were converted to percentage infection before analysis.

### Mixture proportion assessment

Three pairs of spring barley cultivars were sown to assess the effects of different proportions on disease. The pairs were chosen to represent available Recommended List (RL) entries at the time of trialling^44^ with different disease resistance ratings for rhynchosporium (in brackets), namely Optic (4) and Westminster (8), Concerto (4) and Quench (8), and Optic and Waggon (3). For each pair of cultivars mixtures proportions ranged from 100:0 through 95:5, 90:10, 80:20, 65:35, 50:50, 35:65, 20:80, 10:90, 5:95 to 0:100, a total of 11 comparisons for each pair. The performance of each mixture was expressed relative to the proportion of its component cultivars. Thus, for a mixture of 90% cultivar A and 10% cultivar B the expected was the weighted mean calculated as (A*0.9) + (B*0.1). The three different pairs effectively provide replication for the proportions comparisons.

A set of contrasts comparing the response of a mixture plot with that of the corresponding pure stands weighted according to their proportion in the mixture plots, were computed for all mixtures and appropriate sub-sets. The significance of the sum of squares associated with each contrast was tested by comparing it with the residual sum of squares using an F test^45^.

### Modelling the expected effect of mixing proportions in mixtures

Disease intensity within a mixture was modelled as the sum of spread within mixture components, using a pairing of logistic differential equations for temporal and spatial population dynamics:

$$y = \mathop \sum \limits_{i} \frac{{f_{i} }}{{1 + A_{i} \cdot \exp (b_{i} L_{i} - r_{i} t)}}$$

where *y* = disease intensity (which ranges from 0 to 1); *f*_*i*_ = proportion of mixture component *i*; *A*_*i*_ = (*f*_*i*_* − *y_0_)/y_0_, where y_0_ is the initial disease intensity (set to 0.01); *b*_*i*_ = rate of spatial spread in mixture component *i*; *L*_*i*_ = mean nearest neighbour distance for mixture component *i*, calculated as 0.5/√(* f*_*i*_·*d*)^46^, where *d* is the planting density (set to 360 m^−2^); *r*_*i*_ = apparent infection rate in mixture component *i*; and *t* = time (set to 100 days).

This is a classical pairing in theoretical epidemiology and was recently used to extend the theoretical foundation of the ‘Dispersal Scaling Hypothesis’^28^ from spatial to spatiotemporal patterns of disease spread^30^. The model was fitted to the disease data for each of the three mixture proportion assessments in the trial described above.

### Informed consent

All authors consent to this submission.

## Results

### Disease

The proportions trial developed normally (Table 1) and the only disease that occurred at more than trace levels was rhynchosporium. This developed quite late (GS59) and was assessed on 16th and 24th July. Analysis was carried out on the average of these two scores. Optic, Concerto and Westminster gave disease levels in the expected order but Waggon was more resistant and Quench more susceptible than expected from their RL ratings, as was found in later years (AHDB archived RL data).

**Table 1:** Mean disease of spring barley monocultures.

Cultivar	Rhynchosporium (%)	Rhynchosporium rating^b^
Optic(We)^a^	17.88	4
Westminster	1.35	8
Concerto	7.25	4
Quench	14.75	8
Optic(Wa)^a^	12.50	4
Waggon	0.59	3

^a^Optic(We) and Optic(Wa) are 100% Optic plots using the seed used in the Westminster and Waggon mixture series respectively.

^b^AHDB^44^ Recommended List 2011–12.

### Component proportion effect on disease

**Figure 2:**
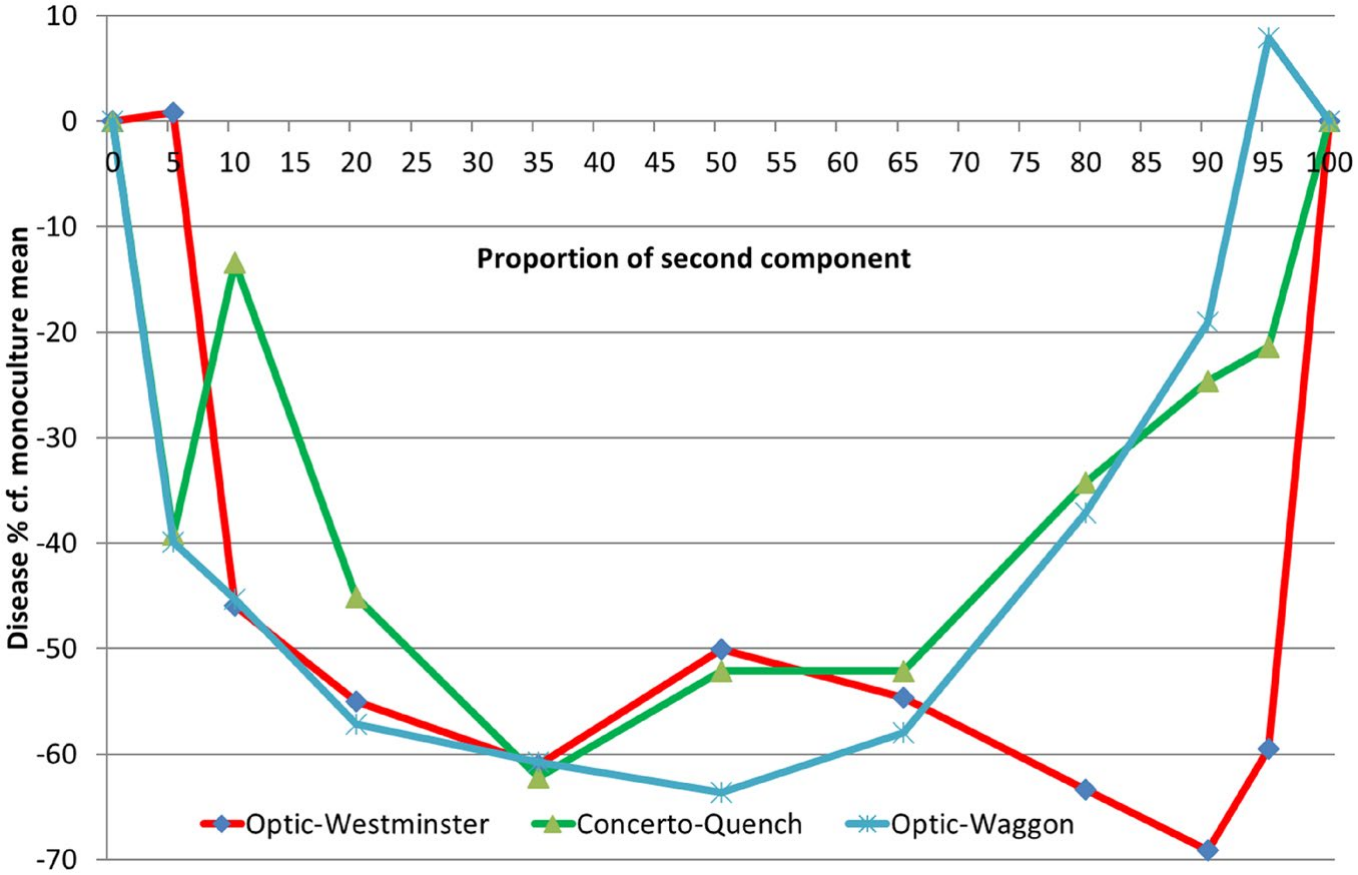
Disease reduction compared with the weighted component monoculture mean for three replacement series of 2-component mixtures no fungicide treatment.

Rhynchosporium symptoms in mixtures ranged from + 8 to − 69% of the mean of the components grown in monocultures. The only two positive scores (+ 1% and + 8%) were with two of the six 95%:5% combinations. All other Rhynchosporium symptoms in mixtures were less than their component mean. The relationship between component proportion and symptom reduction was similar in all three series of component mixtures but it was neither linear nor symmetrical (Fig. 2). For example, the average 25% reduction is achieved with 95:5 proportions, increasing to 35% with 90:10 proportions and 49% with 80:20. However, the maximum reduction was achieved at 58% with proportions of 65:35. Thus a disproportionate decrease in symptoms is achieved by smaller amounts of the added cultivar. Whilst this effect applied to all the cultivars tested, which one has the greater impact seems to depend upon the pairing and was not obviously related to resistance level.

### Modelling the expected effect of mixing proportions in mixtures

**Figure 3:**
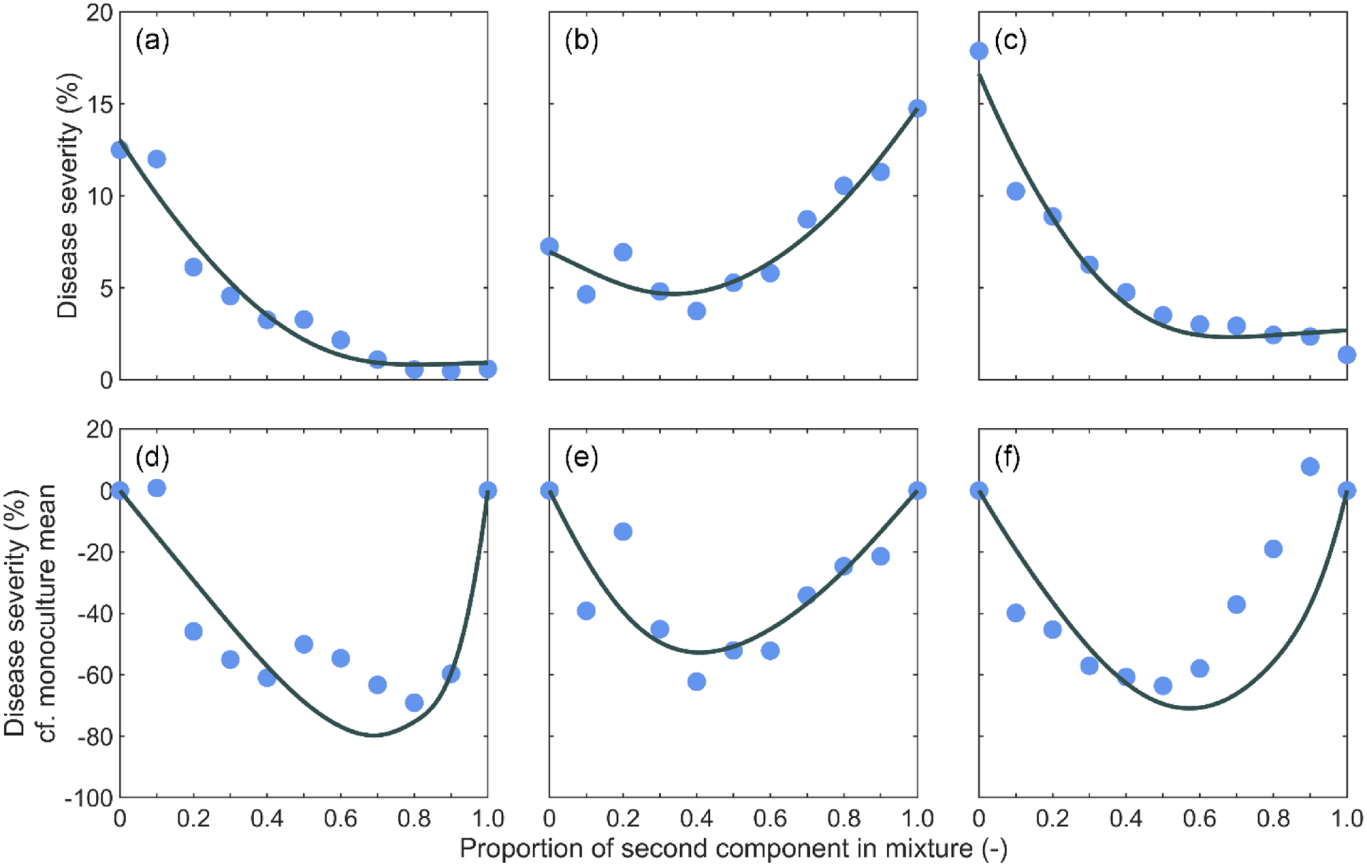
Rhynchosporium disease severity in 2-component barley mixtures. Panels show observed (markers) and predicted (lines) disease severity for (**a**) Optic-Westminster, (**b**) Concerto-Quench, and (**c**) Optic-Waggon, and observed and predicted mixture performance for (**d**) Optic-Westminster, (**e**) Concerto-Quench, and (**f**) Optic-Waggon.

Model fit was excellent, with R^2^ = 0.95, 0.93 and 0.96 for the three pairs of spring barley cultivars (Table 2; Fig. 3a–c). The model was able to describe the disproportionate effect of mixing proportions on disease spread, and predicted mixture performance closely matched observed mixture performance for the first two pairs of spring barley cultivars and reasonably well for the third, with a mean absolute error of 12.26%, 6.89% and 22.26%, respectively (Fig. 3d–f).

**Table 2:** Parameter estimates of the model fit to disease severity data in two-component barley mixtures.

Mixture	b_1_	b_2_	r_1_	r_2_	R^2^
1	41.5 (30.2–62.2)	203.3 (172.6–239.5)	0.01 (0.008–0.01)	0.08 (0.07–0.09)	0.95
2	152.6 (122.6–201.2)	107.1 (43.1–162.8)	0.07 (0.06–0.08)	0.05 (0.03–0.06)	0.93
3	33.0 (11.1–43.7)	266.4 (225.7–313.2)	0.02 (0.01–0.02)	0.10 (0.09–0.11)	0.96

Numbers in parantheses are delete-one jackknife confidence intervals.

## Discussion

Few cereal mixtures trials have investigated different ratios of components but they generally reflect the trait means of their component proportions with a few, such as lodging resistance, exceeding the means^47^. In other trials where mixtures are generally composed of equal proportions of varying numbers of components, many are shown to reduce disease levels below that expected from the mean of the components^1^. Greater reductions are associated with increased number of components in the mixture, clearly shown for the disease reported here too^11^. Here we find rhynchosporium disease reductions of a similar order of magnitude at equal proportion of the components, though achieved even with just two components in the mixture. In addition, we find that a large part of this disease reduction is achieved with even the most unequal proportions of the mixture components.

In general models of disease in cultivar mixtures show that most of the reduction in epidemic rates are attributable to reduction of the density of susceptibles^48^ and that the greater the degree of mixing and the smaller the genotype-unit area the greater the mixture response. However, most models not only assume race-specific resistance, but also generally assume very regular patterns and equal proportions of components. When race non-specific interactions are modelled, the behaviour can be very different leading to new deployment strategies. When random distribution of race non-specific components was compared with regular formats the behaviour patterns changed and greater levels of disease reduction could occur^49^. Irregular behaviour can also occur as the proportions near the extremes. Marshall et al.^50^ found that the behaviour of mixtures was often uneven between two components, with disproportionately large effects on disease reduction towards mixtures containing only a small proportion of one of the components. Sapoukhina et al.^51^ showed that for biotrophic pathogens such as *Puccinia striiformis*, judicious deployment of quantitative resistance in two- or three-component mixtures makes it possible to reduce disease severity using only small proportions of the highly resistant cultivar. This demonstrated that components of mixtures contribute quantitatively differentially to disease control and therefore could be used in different proportions to achieve optimum reductions in disease.

Noting these possible disproportionate behavioral effects on disease spread from small proportion components, the field trial was set up to determine whether they occur with commercial cultivars in practice. Three different pairings showed this behaviour towards the small proportion at one or other extreme whilst the more equal proportion component mixtures gave a smoothed response curve. The skew at the extremes was generally asymmetric showing that one component was generally expressing the effect more strongly than the other. This was characteristic behaviour of the proportion models shown by Marshall et al.^50^ and the three different pairings within the reported trial provided internal validation of this behaviour in the field.

The model presented here also mimics such behaviour. It captured the disproportionate effects of low and high levels of mixing on disease spread, resulting in a u-shaped scaling distribution of mixture performance. It also captured the asymmetry in the scaling distribution resulting from differential trait effects, i.e., differences in resistance, canopy architecture etc. Overall, there was good agreement between predictions and observations, and this study therefore serves as empirical evidence to support the DSH as a conceptual model for spatial and spatiotemporal spread in heterogenous host habitats, such as disease dynamics in crop mixtures, and a tool to predict the proportion of mixing at which mixture performance is maximized.

The overall performance of a mixture is best shown by yield, but yield is a complex character especially in a very plastic response crop such as barley, so there may be many processes of competition and facilitation behind these observations^54^. Disease control will always be an important component of this and the potential to include cultivars with strongly-expressed beneficial traits such as disease control in cultivar mixtures where their benefit can be exploited without diluting the yield- or quality-driving effect of a dominant cultivar is clearly demonstrated, offering such an approach as another valuable tool in the Integrated Pest Management (IPM) toolbox for major cereal crops.

## Data Availability

The data are available from the corresponding author on reasonable request.
